# Changing the Geometry of Representations: *α*-Embeddings for NLP Tasks

**DOI:** 10.3390/e23030287

**Published:** 2021-02-26

**Authors:** Riccardo Volpi, Uddhipan Thakur, Luigi Malagò

**Affiliations:** 1 Romanian Institute of Science and Technology (RIST), 400022 Cluj-Napoca, Romania; uddhipan.thakur@rist.ro (U.T.); malago@tins.ro (L.M.); 2 Transylvanian Institute of Neuroscience, 400157 Cluj-Napoca, Romania

**Keywords:** word embeddings, *α*-embeddings, information geometry, attention mechanism

## Abstract

Word embeddings based on a conditional model are commonly used in Natural Language Processing (NLP) tasks to embed the words of a dictionary in a low dimensional linear space. Their computation is based on the maximization of the likelihood of a conditional probability distribution for each word of the dictionary. These distributions form a Riemannian statistical manifold, where word embeddings can be interpreted as vectors in the tangent space of a specific reference measure on the manifold. A novel family of word embeddings, called α-embeddings have been recently introduced as deriving from the geometrical deformation of the simplex of probabilities through a parameter α, using notions from Information Geometry. After introducing the α-embeddings, we show how the deformation of the simplex, controlled by α, provides an extra handle to increase the performances of several intrinsic and extrinsic tasks in NLP. We test the α-embeddings on different tasks with models of increasing complexity, showing that the advantages associated with the use of α-embeddings are present also for models with a large number of parameters. Finally, we show that tuning α allows for higher performances compared to the use of larger models in which additionally a transformation of the embeddings is learned during training, as experimentally verified in attention models.

## 1. Introduction

Word embeddings are used as a compact representation for the words of a dictionary. They are learned starting from one hot encodings by maximizing the likelihood of a chosen probabilistic model. Rumelhart et al. [1] first introduced the idea of using the internal representation of a neural network to construct a word embedding. Bengio et al. [2] employed a neural network to predict the probability of the next word given the previous ones. Mikolov et al. [3] proposed the use of a recurrent language model based on recurrent neural networks, to learn the vector representations. More recently, this approach has been exploited further and with great success by means of bidirectional LSTM (long short-term memory networks) [4] and transformers [5,6,7].

In this paper, we focus on Skip-Gram (SG), a well-known log-linear model for the conditional probability of the context of a given central word. Together with the continuous bag of words (predicting the central word given the context instead), SG has been shown to be able to efficiently capture syntactic and semantic information [8,9]. Skip-Gram is at the basis of many popular word embedding algorithms such as Word2Vec [8,9] and models based on weighted matrix factorization of the global co-occurrences such as GloVe [10], cf. Levy and Goldberg [11]. These methods are deeply related, Levy and Goldberg showed how Word2Vec SG with negative sampling is effectively performing a matrix factorization of the shifted positive pointwise mutual information [11].

Mikolov et al. [12] noted how, once the embedding space has been learned, syntactic and semantic analogies between words translate into linear relations between the respective word vectors. There have been numerous works investigating the reason for the correspondence between linear properties and word relations. Pennington et al. gave a very intuitive explanation of this behavior in their paper on GloVe [10]. More recently, Arora et al. [13] investigated this property by introducing a hidden Markov model, under some regularity assumptions on the distribution of the word embedding vectors, cf. [14].

Word embeddings are often used as input for other computational models, to solve more complex inference tasks. The evaluation of the quality of a word embedding, which ideally should encode syntactic and semantic information, is not easy to be determined, and different approaches have been proposed in the literature. This evaluation can be in terms of performance on intrinsic tasks such as word similarity [10,15,16,17] or by solving word analogies [8,12]. However, several authors [18,19] have shown a low degree of correlation between the quality of an embedding for word similarities and analogies on one side and on downstream tasks on the other, for instance on classification or prediction, to which the embeddings are given in input. This observation points out the need for a complete experimental evaluation of word embeddings in both intrinsic and extrinsic tasks.

Several works have highlighted the effectiveness of post-processing techniques [15,16], such as Principal Components Analysis (PCA) [14,20], focusing on the fact that certain dominant components are not carriers of semantic nor syntactic information, and thus they act like noise for determinate tasks of interest. Recently, we have proposed in [21,22] a different approach which acts on the learned vectors after training, similarly to a post-processing step, by using a geometrical framework based on Information Geometry [23,24], in which word embeddings are represented as vectors in the tangent space of the probability simplex. A family of word embeddings called natural α-embeddings is introduced, where α is a deformation parameter for the geometry of the probability simplex known in Information Geometry in the context of α-connections. Noticeably, α word embeddings include the standard word embeddings as a special case for α=1. In this paper, we revisit the natural α-embeddings and evaluate them over different tasks. We show how the α parameter provides an extra handle that, by deforming the word embeddings, allows for an improvement of the performance on different intrinsic and extrinsic tasks in Natural Language Processing (NLP). Recently, the use of Riemannian methods has attracted considerable interest in the literature of NLP, recent applications of Riemannian optimization algorithms can be found in [25,26]. In particular, approaches learning word embeddings on a Riemannian manifold have been devised, such as the Poincaré GloVe [27,28] on the Poincaré disk and the Joint Spherical Embeddings (JoSE) [29] on the sphere.

This article is an extended version of [30] and is organized as follows. In Section 2, we introduce the word embeddings based on conditional models, while in Section 3, we review the geometrical framework for α-embeddings. In Section 4, we assess the impact of α-embeddings on the performances of different intrinsic and extrinsic tasks in NLP, with particular emphasis on attention mechanisms, where we show that α-embeddings (controlled by a single scalar) are able to provide better performances than transformations of the embeddings requiring a large number of parameters. Finally, in Section 5, we conclude the paper and present future perspectives.

## 2. Word Embeddings Based on Conditional Models

One of the simplest models which can be used for the unsupervised training of a set of word embeddings are linear conditional models. The Skip-Gram conditional model [9,10] allows the unsupervised training of a set of word embeddings by predicting the conditional probability of any word χ to be in the context of a central word *w*

(1) $$p\left(\chi |w\right)=p_{w}\left(\chi \right)=\frac{\exp(u_{w}^{T}v_{\chi })}{Z_{w}}$$

where $Z_{w}=\sum_{\chi^{\prime }\in D}\exp\left(u_{w}^{T}v_{\chi^{\prime }}\right)$ is the normalization constant. This model is defined by two column vectors $u,v\in R^{d}$ to each word. The set of vectors $u_{w},v_{w}$ for w∈D arranged by rows compose two n×d matrices U,V, respectively. Such matrices are typically learned from data by maximum likelihood estimation [8,10,11].

Equation (1) represents an over-parametrized exponential family in the open n−1 dimensional simplex $P^{n}$, parametrized by two matrices *U* and *V* of size n×d, where *n* is the cardinality of the dictionary D and *d* is the size of the embeddings. Notice that the number of free parameters (2dn) is greater than the number *n* of sufficient statistics ${𝟙}_{\chi }$, corresponding to the one hot encoding of the words of the dictionary. We will refer to the columns of the matrix *V* as $V_{k}$ and to its rows as $v_{\chi }$, seen as column vectors. Analogous notation will be used for *U*. It is common practice in the literature of word embeddings to consider $u_{w}$ or alternatively $u_{w}+v_{w}$ as embedding vectors for a word *w* from the dictionary, see [8,9,10,16,20]. In the remaining part of this section we will review the natural α-embeddings and limit embeddings originally proposed in [21,22] based on notions of Information Geometry [23,24].

After the inference procedure for the estimation of the model parameters, the matrices *V* and *U* are fixed. For each word *w*, the conditional model $p_{w}\left(\chi \right)$ from Equation (1) is a *d*-dimensional exponential family $E_{V}$ in the n−1 dimensional open simplex $P^{n}$, which models the probability of a word χ in the context of the central word *w*. From this perspective, the exponential model $E_{V}$ has *d* sufficient statistics corresponding to the columns of *V*, while each row $u_{w}$ of *U* corresponds to an assignment for the natural parameters, which identifies a probability distribution in the model. During training, both matrices *U* and *V* are updated to maximize the likelihood of the observed data in the corpus. This implies that both the sufficient statistics of the exponential model $E_{V}$ are updated, by changing the columns of *V*, as well as the assignment of the natural parameters $u_{w}$ of each conditional distribution $p_{w}$.

**Figure 1:**
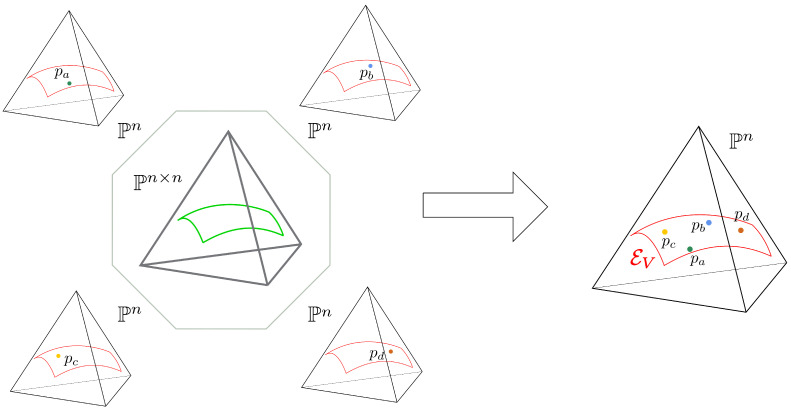
The Skip-Gram model is defines a joint curved model in the (n×n−1)-dimensional simplex. Some faces of this model correspond to the conditional models p(χ|w) for some *w*. The conditional models are defined over the same sample space and have the same sufficient statistics determined by *V*, they represent, in fact, different points on the same exponential family $E_{V}$ embedded in $P^{n}$. At each training step, the model $E_{V}$ varies with *V*.

Each conditional model p(χ|w) lies inside a face of the (n×n−1)-dimensional simplex, corresponding to the ambient space for the joint distribution p(χ,w) parametrized by U,V. Since the conditional models are defined over the same sample space and have the same sufficient statistics determined by *V*, we can identify them with a single exponential family $E_{V}$ embedded in $P^{n}$, as depicted in Figure 1.

## 3. α-Embeddings

In Information Geometry, a statistical model is represented as a Riemannian manifold endowed with a Riemannian metric given by the Fisher information matrix [23,24,31]. The Fisher matrix for the exponential family (1) corresponds to the covariance matrix of the centered sufficient statistics

(2) $$I\left(p_{0}\right)=E_{p_{0}}\left[\Delta v_{\chi }\left(p_{0}\right)\Delta v_{\chi }{\left(p_{0}\right)}^{T}\right]=\Delta V{\left(p_{0}\right)}^{T}\mathrm{diag}(p_{0})\Delta V\left(p_{0}\right),$$

where $\Delta V\left(p_{0}\right)=\left(V-E_{p_{u}}\left[V\right]\right)$ are the centered sufficient statistics evaluated over the dictionary and $\Delta v_{\chi }\left(p_{0}\right)$ corresponds to a row of $\Delta V(p_{0})$ expressed as a column vector.

The geometry of a statistical manifold defined by a metric and a connection can be induced by a divergence [23]. Taking two positive measures *p* and *q*, the family of α-divergences, for α∈R, are defined as

(3) $$D^{\left(\alpha \right)}\left[p||q\right]=\left\{\begin{array}{cc} \frac{4}{1-\alpha^{2}}\left(\frac{1-\alpha }{2}\sum_{i}p_{i}+\frac{1+\alpha }{2}\sum_{i}q_{i}-\sum_{i}p_{i}^{\frac{1-\alpha }{2}}q_{i}^{\frac{1+\alpha }{2}}\right)\;\; & \alpha \neq \pm 1\\ \sum_{i}p_{i}-\sum_{i}q_{i}+\sum_{i}q_{i}\ln\frac{q_{i}}{p_{i}}\;\; & \alpha =-1\\ \sum_{i}q_{i}-\sum_{i}p_{i}+\sum_{i}p_{i}\ln\frac{p_{i}}{q_{i}}\;\; & \alpha =+1. \end{array}\right.$$

It is a known fact that α-divergences are also f-divergences and thus induce the same metric on the manifold, which is the Fisher metric [32], indeed by taking the Hessian of an α-divergence between infinitesimally close probability distributions, we obtain the Fisher information matrix $I(p_{0})$ for any α. The exponential family, endowed with the family of α-divergences is a dually-flat manifold, meaning that the α-divergences define a corresponding family of α-connections [23], which are dually coupled with respect to the metric. For α=0, we obtain the Levi–Civita connection, which is by definition compatible with the metric and thus self-dual. It is possible to prove that the $h_{\alpha }$-representation

(4) $$h_{\alpha }\left(p\right)=\left\{\begin{array}{cc} \frac{2}{1-\alpha }p^{\frac{1-\alpha }{2}} & \alpha \neq 1\\ \log p & \alpha =1, \end{array}\right.$$

provides a parametrization in which the corresponding α-connection is flat.

In our previous papers [21,22], using an information geometric framework, we have introduced a novel family of embeddings called natural α-embeddings. Given a reference measure $p_{0}$ in the exponential family $E_{V}$, the natural α-embedding of a given word *w* from the dictionary is defined as the α-projection $\Pi_{p_{0}}^{\left(\alpha \right)}$ of the α-logarithmic map ${\mathrm{Log}}_{p_{0}}^{\left(\alpha \right)}w$ onto the tangent space $T_{h_{\alpha }\left(p_{0}\right)}E_{V}^{\left(\alpha \right)}$ of the model $E_{V}^{\left(\alpha \right)}=h_{\alpha }\left(E_{V}\right)$ represented by means of the $h_{\alpha }$-representation, used to deform probability distributions in the simplex [23,32]. The main intuition behind this definition is that a word embedding for *w* corresponds to the vector in the tangent space of $p_{0}$ that allows to reach $p_{w}$ starting from $p_{0}$. Since the $h_{\alpha }$-representation, the logarithmic map and the projection are expressed as a function of the same parameter α, a family of natural α-embeddings $W_{p_{0}}^{\left(\alpha \right)}\left(w\right)\in T_{h_{\alpha }(p_{0})}h_{\alpha }\left(E_{V}\right)$ can be defined depending on α. In the following, we report the main formula for the computation of the natural α-embeddings, all the detailed derivations can be found in [21,22]. By combining the formula for the α-projection and the α-logarithmic map, we obtained the following formula for the natural α-embeddings

(5) $$\begin{array}{cc} W_{p_{0}}^{\left(\alpha \right)}\left(w\right) & =\Pi_{0}^{\left(\alpha \right)}\left({\mathrm{Log}}_{p_{0}}^{\left(\alpha \right)}p_{w}\right)=I{\left(p_{0}\right)}^{-1}\sum_{\chi }l_{p_{0}\;w}^{\left(\alpha \right)}\left(\chi \right)\;p_{0}\left(\chi \right)\;\Delta v_{\chi }\left(p_{0}\right)\\ & =I{\left(p_{0}\right)}^{-1}\Delta V{\left(p_{0}\right)}^{T}\mathrm{diag}(p_{0})\;l_{p_{0}\;w}^{\left(\alpha \right)} \end{array}$$

where, employing a slight abuse of notation, $p_{0}$ is a vector, $\mathrm{diag}(p_{0})$ is a diagonal matrix with diagonal $p_{0}$, and the vector $l_{p_{0}\;w}^{\left(\alpha \right)}$ is defined with components

(6) $$l_{p_{0}\;w}^{\left(\alpha \right)}\left(\chi \right)=\left\{\begin{array}{cc} \ln p_{w}\left(\chi \right)-\ln p_{0}\left(\chi \right) & \alpha =1,\\ \frac{2}{1-\alpha }\left({\left(\frac{p_{w}\left(\chi \right)}{p_{0}\left(\chi \right)}\right)}^{\frac{1-\alpha }{2}}-1\right) & \alpha \neq 1 \end{array}\right..$$

We summarize the α-embeddings calculation with the following pseudo-code (see in Algorithm 1).
**Algorithm 1:**α-embeddings.
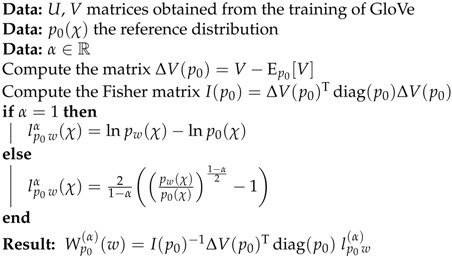


α-embeddings can be used both for downstream tasks and also to evaluate similarities and analogies in the tangent space of the manifold [21,22]. Given two words *a* and *b*, a measure of similarity is defined by the geometric cosine similarity between α-embeddings

(7) $${\mathrm{sim}}_{p_{0}}^{\left(\alpha \right)}\left(a,b\right)=\frac{{\langle W_{p_{0}}^{\left(\alpha \right)}\left(a\right),W_{p_{0}}^{\left(\alpha \right)}\left(b\right)\rangle }_{I(p_{0})}}{{\left|\left|W_{p_{0}}^{\left(\alpha \right)}\left(a\right)\right|\right|}_{I(p_{0})}{\left|\left|W_{p_{0}}^{\left(\alpha \right)}\left(b\right)\right|\right|}_{I(p_{0})}},$$

Moreover, analogies of the form a:b=c:d can be solved by minimizing an analogy measure $\kappa_{p_{0}}^{\left(\alpha \right)}\left(p_{a},p_{b},p_{c},p_{d}\right)$ reducing to the difference between the vectors $W_{p_{0}}^{\alpha }\left(b\right)-W_{p_{0}}^{\alpha }\left(a\right)$ and $W_{p_{0}}^{\alpha }\left(d\right)-W_{p_{0}}^{\alpha }\left(c\right)$ computed in $p_{0}$ with respect to the metric

(8) $${∥W_{p_{0}}^{\left(\alpha \right)}\left(b\right)-W_{p_{0}}^{\left(\alpha \right)}\left(a\right)-W_{p_{0}}^{\left(\alpha \right)}\left(d\right)+W_{p_{0}}^{\left(\alpha \right)}\left(c\right)∥}_{I(p_{0})}.$$

It has been shown in [21,22] that for α=1, if $p_{0}$ equals the uniform distribution over the dictionary, the embeddings of Equation (5) reduce to the standard vectors $u_{w}$. Furthermore, by substituting the Fisher information matrix $I(p_{0})$ with the identity matrix, Equations (7) and (8) reduce to the standard formulas used in the literature for similarities and analogies [8,9,10]. Proposition 3 in [21] or equivalently Proposition 1 in [22] provides conditions under which the Fisher information matrix is isotropic, i.e., proportional to the identity.

It is quite common practice in the literature to use the embedding vectors u+v, which have been shown to provide better results [10] than simply *u* vectors. In the context of natural α-embeddings, the vectors u+v can be interpreted as a shift of the natural parameters *u* of the exponential family. It can be demonstrated [21,22] that this corresponds to a reweighting of the probabilities in Equation (1)

(9) $$p^{\left(+\right)}\left(\chi |w\right)=\frac{1}{N_{w}}\exp\left(u_{w}^{T}v_{\chi }\right)p\left(\chi |w\right),$$

in which $N_{w}$ is an additional factor emerging from the normalization. Equation (9) represents a change of reference measure proportional to $\exp(u_{w}^{T}v_{\chi })$, i.e., giving more importance to those words χ whose *v* vectors are aligned to that of the central word *w*. This defines an analogous notion of u+v embeddings (popularly used in the literature) in the context of α-embeddings

(10) $${\tilde{W}}_{p_{0}}^{\left(\alpha \right)}\left(w\right)=\Pi_{p+0}^{\left(\alpha \right)}\left({\mathrm{Log}}_{p_{0}}^{\left(\alpha \right)}p^{\left(+\right)}\left(\cdot |w\right)\right).$$

### Limit Embeddings

The behavior of the α-embeddings for α progressively approaching minus infinity turns out to be of particular interest. Indeed, in this case, $l_{p_{0}\;w}^{\alpha }\left(\chi \right)$ is progressively more and more peaked on the words χ which have larger ratio $p_{w}\left(\chi \right)/p_{0}\left(\chi \right)$, up to the point of corresponding to a delta distribution over the set

(11) $$\chi_{w}^{*}=\underset{\chi }{\arg\max}\frac{p_{w}\left(\chi \right)}{p_{0}\left(\chi \right)}.$$

Notice that the norm of $l_{p_{0}\;w}^{\alpha }\left(\chi \right)$ tends to infinity as α tends to minus infinity, since 1−α tends to infinity and thus the maximum of the probability ratio (which is always greater or equal to 1 for any two distributions) is progressively predominant, see Equation (6). Since for all tasks of interest, we always use normalized α-embeddings (either with the identity matrix or with the Fisher metric), this allows us to consider only the direction of the tangent vectors. In the limit of α going to minus infinity, the un-normalized limit embeddings simplify to

(12) $$\begin{array}{cc} LW_{p_{0}}^{\left(\alpha \right)}\left(w\right) & =\lim_{\alpha \to -\infty }W_{0}^{\left(\alpha \right)}\left(w\right)\\ & =I{\left(p_{0}\right)}^{-1}\Delta V{\left(p_{0}\right)}^{T}\mathrm{diag}(p_{0})\;{\underline{1}}_{\chi_{w}^{*}}. \end{array}$$

where ${\underline{1}}_{\chi_{w}^{*}}$ is the indicator function for the words in $\chi_{w}^{*}$ from the dictionary. Notice that $\mathrm{diag}p_{0}$ weights the rows of ΔV, while the indicator function ${\underline{1}}_{\chi_{w}^{*}}$ selects only a restricted number of rows of the matrix, which are then premultiplied by the inverse of the Fisher information matrix. In most cases, the ratio has a unique argmax, hence the limit embeddings depend on one single row of ΔV

(13) $$LW_{p_{0}}^{\left(\alpha \right)}\left(w\right)=p_{0}\left(\chi_{w}^{*}\right)I{\left(p_{0}\right)}^{-1}\Delta v_{\chi_{w}^{*}}\left(p_{0}\right).$$

This simple formula allows the straightforward implementation of geometrical methods which are based on un-normalized α-embeddings in the limit case of α going to minus infinity. Additionally, let us notice that in the case for two words $w^{\prime }$ and $w^{\prime \prime }$, we have $\chi_{w^{\prime }}^{*}=\chi_{w^{\prime \prime }}^{*}$, then the associated α-embeddings will tend to correspond as α→−∞, thus limit embeddings also naturally induce a clustering in the embedding space.

## 4. Experiments

We considered two corpora: the English Wikipedia dump October 2017 (enwiki), with 1.5B words, and its augmented version composed by Gutenberg [33], English Wikipedia and Book-Corpus [34,35,36] (geb), with 1.8B words. We used the WikiExtractor Python script [37] to parse the Wikipedia dump xml file. A minimal preprocessing was performed, by lowercasing all the letters and removing stop-words and punctuation.

For each corpus, we trained a set of word embeddings with vector sizes of 50 and 300. We employed a cut-off minimum frequency (m0) of 1000, obtaining a dictionary of about 67 k words for both enwiki and geb. For GloVe, we used the code at [38], the window size was set to be 10 as in [10], with a decaying weighting rate from the central word of 1/d for the calculation of co-occurrences. We trained the models for a maximum of 1000 iterations. For Word2Vec SG, we used the code at [39] with window 10 and negative sampling 5. We trained the models for 100 epochs.

The embeddings in Equation (5) will be denoted with E in all the figures and tables from this section, while the limit embeddings in Equation (12) will be denoted with LE. Embeddings have been normalized either with the Fisher information matrix (F) or the identity matrix (I). Similarly, scalar products will be computed either with the Fisher information matrix (F) or the identity matrix (I). In the following, in case both inner product and normalization are used in the same experiment, they will be computed with respect to the same metric (either F or I). For the reference distribution needed for the computation of the α-embeddings, we have chosen the uniform distribution (0), the unigram distribution of the model (u) obtained by marginalization of the joint distribution learned by the model

$$p\left(w\right)=\sum_{\chi }\frac{\exp(u_{w}^{T}v_{\chi })}{Z},$$

or the unigram distribution estimated from the corpus (ud). Embeddings are denoted by U if in the computation of Equations (5) and (12) the formula used for $p_{w}$ is Equation (1), while they will be denoted by U+V if Equation (9) is used instead.

We evaluate the α-embeddings on intrinsic tasks such as similarities, analogies, and concept categorization, as well as on extrinsic ones like document classification, sentiment analysis, and sentence entailment. We consider α with step 0.1 between (–10, 10) for similarities and analogies. We perform experiments with step 0.1 for α∈ (–2, 2), step 0.2 for α∈ (–10, –2) ∪ (2, 10) and step 1 for α∈ (–30, –10) ∪ (10, 30) for concept categorization, document classification, sentiment analysis, and sentence entailment.

### 4.1. Similarities, Analogies, and Concept Categorization

**Figure 2:**
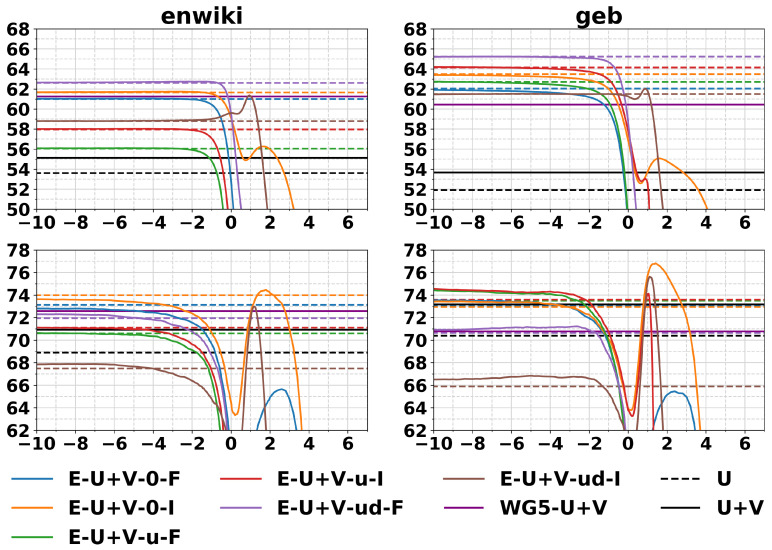
Word similarities expressed in Spearman correlation × 100 (**top**) and word analogies accuracies (**bottom**) for different values of α. The left column reports experiments on enwiki, while the right column reports experiments on geb. U, U+V, and WG5-U+V are the GloVe vectors of size 300 described in the text, centered and normalized. Figure from [30].

In Figure 2, we report results for similarities and analogies with embeddings of size 300. For similarities, we consider the following datasets: ws353 [40], mc [41], rg [42], scws [43], men [44], mturk287 [45], rw [46], and simlex999 [47]. For analogies, we use the Google analogy dataset [8] split, as is common practice in the literature, in semantic analogies (sem) and syntactic analogies (syn), or alternatively considering all of them (tot). The limit embeddings (colored dotted lines) achieve good performances on both tasks, above the competitor methods from the literature U and U+V based on GloVe vectors centered and normalized by column, as described in Pennington et al. [10]. Comparison with baseline methods from the literature on word similarity is presented in Table 1, we compared with the limit embeddings since they usually seem to perform well on the similarity task, see Figure 2 top row. The limit embeddings methods reported in the table outperform the Wiki Giga 5 pretrained vectors [10] (6B words corpus) and other comparable baselines from the literature.

In Table 2, we report the best performances for the analogy task on α-embeddings, where α is selected with cross-validation. For the syn dataset, using the embeddings trained on the enwiki corpus, the limit embeddings have been found to work better instead. The standard deviations reported are obtained by averaging the performances on test of the top three α selected on the basis of the best performances on validation. The standard deviations obtained are relatively small, which indicates that tuning α is easy also on tasks with small amounts of data in cross-validation. The best tuned α on the geb dataset outperform the baselines for all experiments.

**Figure 3:**
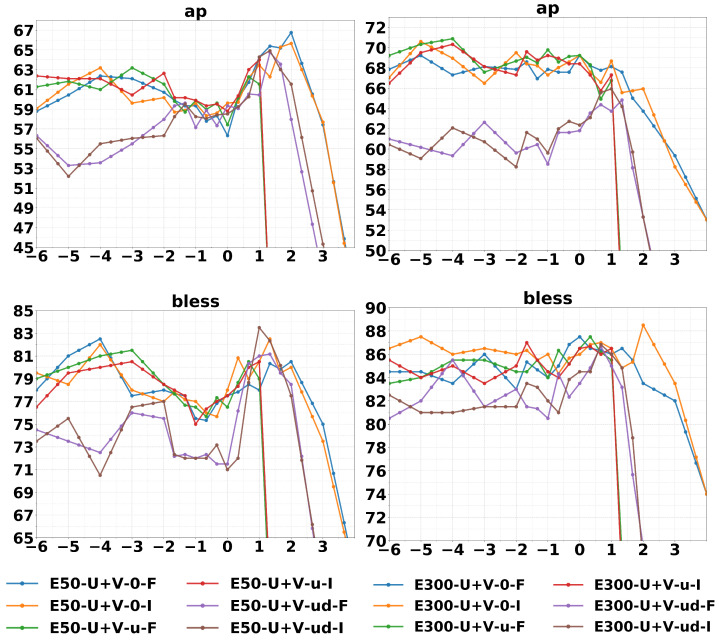
Cluster purity on concept categorization task (plotted with 3-point average). Figure from [30].

The last intrinsic tasks considered are cluster purity for concept categorization datasets AP [49] and BLESS [50]. For each dataset and for each set of embeddings, we run a spherical k-means algorithm with the help of the Python package spherecluster [51,52]. More specifically, we normalize the embeddings in the tangent space $T_{h_{\alpha }\left(p_{0}\right)}E_{V}^{\left(\alpha \right)}$ to obtain points on a sphere embedded in the tangent space itself, and then we compute distances on such sphere with the arccosine of the cosine similarity in Equation (7). We set n_init = 300, n_clusters equal to the number of groups of the dataset, and use default parameters otherwise. We run the clustering algorithms 10 times and we select the best results. In Table 3, we report clusters purity on the geb word embeddings of dimension 300. Tuning the value of α allows us to obtain a considerable cluster purity improvement with respect to the standard GloVe baseline (GloVe U+V). Interestingly, we notice how the purity values are superior to the values reported in the literature and comparable only with Baroni et al. [48], where the authors employ a hyperparameter tuning for the training of GloVe. The purity curves (Figure 3) are more noisy w.r.t. similarities and analogies, this is because the datasets available for this task are quite limited in size. Almost all curves exhibit a peak, which is relatively more pronounced for smaller embedding sizes, while the limit behavior for large negative α performs better for a larger embedding size. This points to the fact that clustering induced by the limit embeddings of Equation (12) is better behaved when the dimension of the embeddings, and the number of sufficient statistics, is larger.

### 4.2. Document Classification and Sentiment Analysis

In this subsection, we present results on the 20 Newsgroup multi-classification [54] and the IMDBReviews sentiment analysis [55]. The α-embeddings are normalized before training either with I or F. We use a linear architecture (BatchNorm+Dense) for both tasks, while for sentiment analysis we also use a recurrent architecture (Bidirectional LSTM 32 channels, GlobalMaxPool1D, Dense 20 + Dropout 0.05, Dense). When using the linear architecture, a continuous bag of words representation is used. In Table 4 and Table 5, we report the best α chosen with respect to the validation set and the best performance for the limit embeddings of size 300. Limit embeddings have been generalized, by considering the words associated to the *t* largest values for the probabilities ratio in Equation (11), instead of a single one. We denote this modification by -t1/3/5. Furthermore, we indicate with -w the experiments in which the $\chi^{*}$ rows of ΔV in Equation (12) are weighted with $p_{w}\left(\chi \right)/p_{0}\left(\chi \right)$, instead of $p_{0}\left(\chi \right)$). The improvements reported in Table 4 and Table 5 are small but appear on every task (at least 0.5% in accuracy) on both Newsgroups and IMDBReviews, such increase of performance are present also when network architectures of increased complexity are used, such as for bidirectional LSTM.

**Figure 4:**
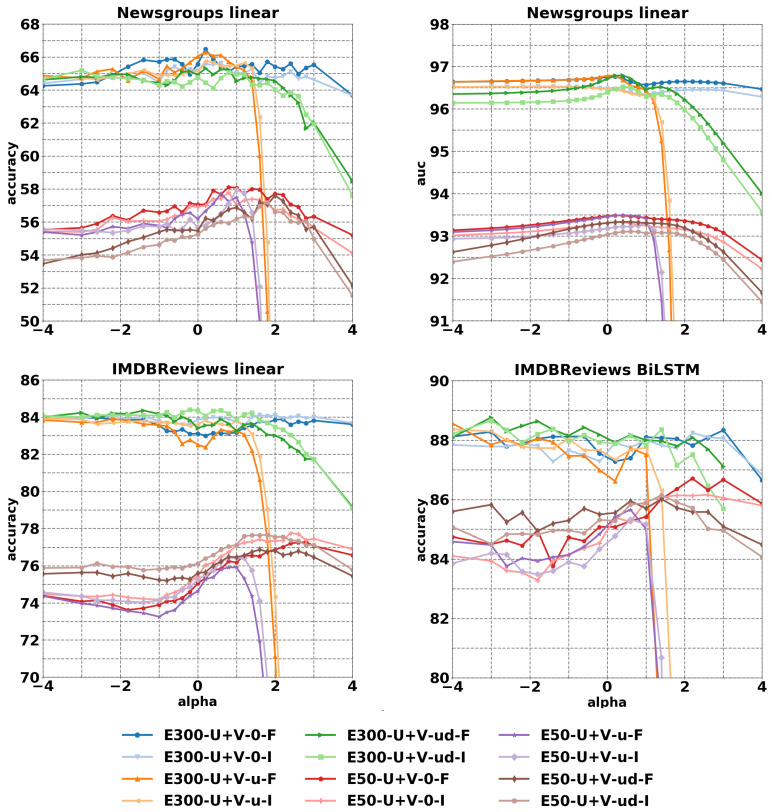
Accuracy and AUC on 20 Newsgroups and IMDB Reviews datasets for varying α. The metrics I and F refer to the normalization of the embeddings before training. Figure from [30].

Figure 4 reports the curves for the values on test with early stopping based on the validation for embedding sizes of 50 and 300. The improvements when α is tuned are higher on size 50, exhibiting a more evident peak. For size 300 the improvements are smaller but consistent. In particular, a peak performance for α can be always easily identified for a chosen reference distribution and a chosen normalization. 

### 4.3. Sentence Entailment

In this subsection, we evaluate the impact on the performance of α-embeddings on the task of sentence entailment, solved by a neural network with a more complex architecture. We consider the Stanford Natural Language Inference (SNLI) dataset [56], constituted of pairs of sentences (a,b). The task is to predict whether *a* is entailed by *b*, *b* contradicts *a*, or whether their relationship is neutral. To perform the task, we choose the decomposable attention model from Parikh et al. [57], implementing the attention mechanism from Bahdanau et al. [58]. The decomposable attention model breaks the sentence apart into subsections and aligns them to check their similarity or differences, thus determining whether the sentences are entailed or not. The model consists of three trainable components along with a part for input representation: Attend, Compare, and Aggregate. All three components consists of separate neural networks (with attention mechanisms) which are trained jointly. Intra-sentence attention is used in the case we implemented.

The model was trained as follows. The batch size was set to 32 and the dropout ratio used before all the was ReLU layers fixed to 0.2. Batch normalization was used in the attention layers to ensure robustness and faster convergence. The learning rate was set to 0.05, along with a decay rate of 0.1 after every 20 epochs. The experiments were run for 200 epochs, with the Adagrad optimizer. The weights of the network were initialized with a Gaussian distribution with a mean of 0 and a standard deviation of 0.01. We implemented the attention model in PyTorch [59], starting from the code by Kim [60] and Li [61]. In the first step of preprocessing, we removed punctuation and stop-words from the sentences in the dataset. During training, we used a maximum sentence length of 50 words. While using the embeddings, each sentence was tokenized and tokens for padding and unknown words were added. The 300 dimensional geb α-embeddings were used. Each vector was normalized with either the Fisher or the identity matrix. All embeddings remained fixed while training.

Two types of experiments were performed. In one set of experiments, the embedding vectors were linearly transformed by means of a matrix whose entries are learned during training. In the original paper by Parikh et al. [57] such a linear transformation projects the word embeddings to a 200 dimension space, however, we decided to keep the dimensions fixed to 300 to compare the performance with those of the next set of experiments, where no projection matrix is used. In the following, we refer to the linear transformation as a projection matrix.

**Figure 5:**
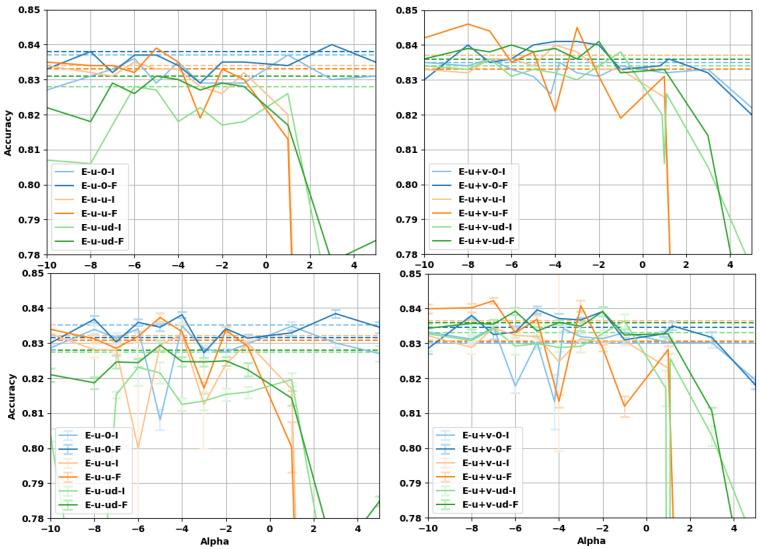
Accuracy of the decomposable attention model over the sentence entailment task without projection matrix. (**top row**) Test accuracies at best validation point during training; (**bottom row**) test accuracies averaged over the last 10 epochs of training. (**Left column**) U embeddings, (**right column**) U+V embeddings. The vectors have been normalized either with the Fisher information matrix (F) or with the identity matrix (I). The limit embeddings are represented by the dashed lines of the corresponding color.

**Figure 6:**
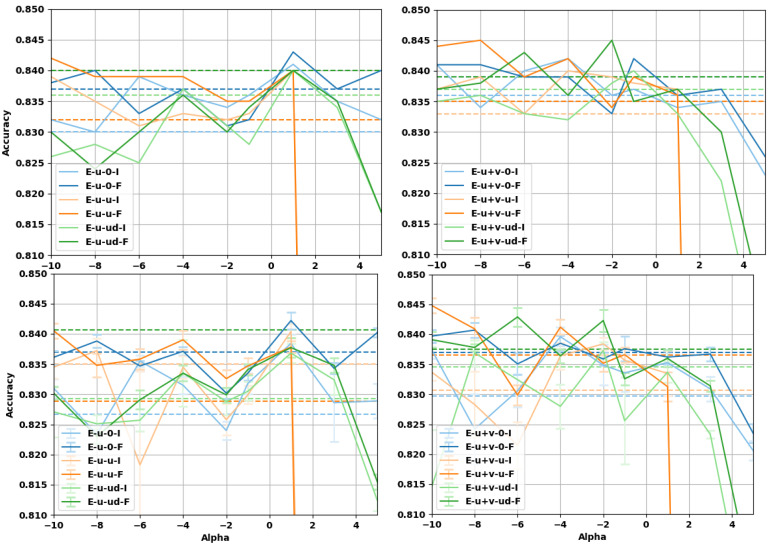
Accuracy of the decomposable attention model with an additional trainable projection matrix. (**top row**) Test accuracies at best validation point during training; (**bottom row**) test accuracies averaged over the last 10 epochs of training. (**left column**) U embeddings, (**right column**) U+V embeddings. The vectors have been normalized either with the Fisher information matrix (F) or with the identity matrix (I). The limit embeddings are represented by the dashed lines of the corresponding color.

The results of the prediction accuracy for the sentence entailment task as a function of α are reported in Figure 5 and Figure 6. For the case with a trainable projection matrix (Figure 6), we observe that the baseline accuracy is higher and the gain deriving from the use of α-embeddings is smaller. This is expected, as the projection matrix already provides a linear transformation of the embedding (task-dependent fine-tuning) before the attention mechanism. It should be noted that using a projection matrix of dimension 300×300 adds about 12.4 percent more trainable parameters to the architecture (which has ≈$7.25\times 10^{5}$ parameters without the projection layer). For the case where α-embeddings are used without the projection matrix, we can see that there is a larger improvement to the accuracy, but the baseline is lower in this case. The projection matrix already provides a linear transformation of the word-vectors limiting the improvement that α-embeddings can have over the baseline. It is worth noticing that α-embeddings always provide an improvement compared to regular embeddings given by α=1, even on the more complex attention model with projection. Interestingly enough, for certain values of α, we can see that the accuracy of the α-embeddings without projection surpasses the baseline values for the same task when the projection is used (and are even comparable with the best α), see Table 6. This points to the fact that using α-embeddings and tuning the value of α can be an alternative to the use of more complicated architectures where a linear transformation of the embeddings is used, reducing the computational efforts and obtaining better performances.

## 5. Conclusions

In this paper, we have evaluated experimentally the performance of α-embeddings on several intrinsic and extrinsic tasks in NLP. For word similarities and analogies, the α-embeddings provide significant improvements over standard embedding methods corresponding to α=1 and over baselines from the literature. Improvements are present on all the tasks tested with different margins, depending on the value of α on the chosen reference distribution (0, u, ud) and the normalization method (I, F). We observe that the best value of α depends both on the task and on the dataset. Thus, α-embeddings provide an extra hyperparameter on the optimization problem when solving the specific task, allowing to choose the best deformation of the space based on data. Values of α lower than 1 and negative seem to be preferred across most tasks. Limit embeddings provide a simple alternative that does not require validation over α but can still offer an improvement on several tasks of interest. Furthermore, limit embeddings induce a clustering in the space of the representations learned by the SG model during training. Performances of the limit embeddings grow with the increasing dimension of the embedding on Newsgroups and IMDB Reviews, pointing to the possibility that limit embeddings show better performances than α-embeddings on higher dimensional spaces.

On the decomposable attention model, the accuracy of α-embeddings without projection surpasses the baseline values for the same task with projection and is also comparable with the best α with projection. This is an indication that using α-embeddings and tuning the value of α can allow to save the extra parameters used to learn a transformation of the embeddings during training, which is costly, reducing the computational efforts and obtaining better performances.

In the present work, α is chosen on the basis of the performance on the validation set. As a future work, we advocate for the design of an automated mechanism optimizing α during training, leading to the definition of an α GloVe loss function and an α attention mechanism. As a future work, we advocate for the design of training algorithms based on α, which are able to automatically tune such hyperparameter and thus learn the best geometry for the task at hand.

## Figures and Tables

**Table 1:** Spearman correlations for the similarity tasks. WG5 denotes the wikigiga5 pretrained vectors on 6B words [10] tested for comparison on the dictionary of the smaller corpora enwiki and geb. U and U+V are the standard methods either for GloVe or Word2Vec. PSM refers to the accuracies reported by Pennington et al. [10] on enwiki, BDK is the best setup across tasks (as a result of hyperparameters tuning) reported by Baroni et al. [48], and LGD are the best methods in cross-validation with fixed window sizes of 5 and 10 (as a result of hyperparameters tuning) reported by Levy et al. [17].

	Method	ws353	mc	rg	scws	ws353s	ws353r	Men	mturk287	rw	simlex999	All
enwiki	LE-U+V-ud-F (our)	75.5	83.4	81.5	63.5	77.8	69.2	75.6	60.1	55.6	41.6	62.6
	GloVe WG5-U+V	65.1	73.8	77.6	62.2	71.3	60.7	77.2	65.7	51.5	41.0	61.3
	GloVe U	60.2	69.3	69.8	58.3	67.1	56.4	69.2	67.2	47.1	31.4	53.6
	GloVe U+V	63.8	74.5	75.2	58.7	69.5	60.9	71.6	67.3	45.5	32.2	55.1
	Word2Vec U	64.7	73.5	78.4	63.6	73.7	56.1	72.9	65.4	47.3	34.5	59.1
	Word2Vec U+V	66.1	75.3	76.1	64.1	75.2	57.3	72.5	63.8	46.1	33.4	58.7
geb	LE-U+V-ud-F (our)	77.0	81.2	83.5	65.0	80.3	68.7	79.6	62.4	59.3	46.9	65.2
	GloVe WG5-U+V	65.1	73.8	77.9	61.8	71.3	60.7	77.2	65.7	53.2	40.6	60.4
	GloVe U	61.3	73.0	76.3	58.7	68.6	54.0	68.7	68.1	48.9	30.6	51.9
	GloVe U+V	64.9	77.4	79.9	59.1	71.5	58.8	71.4	68.1	48.5	32.5	53.7
	Word2Vec U	65.5	77.8	74.7	62.6	73.2	58.5	73.1	67.5	48.3	32.9	59.0
	Word2Vec U+V	69.4	77.4	78.2	63.5	76.0	62.5	73.9	65.3	49.0	32.9	59.6
GloVe PSM 6B [10]		65.8	72.7	77.8	53.9	-	-	-	-	38.1	-	-
Word2Vec BDK [48]		73	-	83	-	78	68	80	-	-	-	-
GloVe LGD win5 [17]		-	-	-	-	74.5	61.7	74.6	63.1	41.6	38.9	-
GloVe LGD win10 [17]		-	-	-	-	74.6	64.3	75.4	61.6	26.6	37.5	-
Poincaré GloVe 100D [28]		62.3	80.5	76.0	-	-	-	-	-	42.8	31.8	-
JoSE 100D [29]		73.9	-	-	-	-	-	74.8	-	-	33.9	-

**Table 2:** Accuracy on analogy tasks for the different methods for enwiki and geb corpora. The best α is selected with a 3-fold cross validation (α between −10 and 10, with step 0.1), unless the limit embeddings is the one performing best. The best α values are reported in parentheses. PSM are the accuracies reported by Pennington et al. [10] on enwiki, BDK is the best setup across tasks (as a result of hyperparameters tuning) reported by Baroni et al. [48].

	Method	Sem	Syn	Tot
enwiki	E-U+V-0-I (our)	84.5±0.4(1.8±0.1)	67.33 (−∞)	74.4±0.1(1.7±0.1)
	GloVe WG5-U+V	79.4	67.5	72.6
	GloVe U	77.8	62.1	68.9
	GloVe U+V	80.9	63.4	70.9
	Word2Vec U	74.58	54.96	63.39
	Word2Vec U+V	75.44	55.03	63.81
geb	E-U+V-0-I (our)	83.8±0.4(1.7±0.1)	72.2±0.4(1.3±0.1)	76.7±0.3(1.3±0.1)
	GloVe WG5-U+V	78.7	65.2	70.7
	GloVe U	75.7	66.8	70.4
	GloVe U+V	80.0	68.5	73.2
	Word2Vec U	71.20	52.62	60.15
	Word2Vec U+V	71.59	51.88	59.87
GloVe PSM 1.6B [10]		80.8	61.5	70.3
GloVe PSM 6B [10]		77.4	67.0	71.7
Word2Vec BDK [48]		80.0	68.5	73.2
Poincaré GloVe 100D [28]		66.4	60.9	63.4

**Table 3:** Clustering purity (×100) with the spherical clustering method described in the main text, compared with numbers from literature. The max, average, and standard deviation are obtained over 10 runs. BDK is the best setup across tasks (as a result of hyperparameter tuning) reported by Baroni et al. [48].

Dataset	Method	Max Purity	Avg Purity
AP	E-U+V-u-F (α = −4)	70.9	66.2 ± 2.1
	GloVe U+V	64.3	61.4 ± 2.5
	Word2Vec U+V	63.5	61.0 ± 1.6
	GloVe [53]	61.4	-
	Word2Vec [53]	68.2	-
	Word2Vec BDK [48]	71.0	-
BLESS	E-U+V-ud-I (α = 1.1)	89.0	83.5 ± 2.6
	GloVe U+V	86.0	83.4 ± 2.5
	Word2Vec U+V	80.0	77.3 ± 2.5
	GloVe [53]	82.0	-
	Word2Vec [53]	81.0	-

**Table 4:** AUC and accuracy on test of 20 Newsgroups multiclass classification (BatchNorm + Dense), compared to baseline vectors. Best α and best limit method (on validation) are reported in parentheses.

Method	20 Newsgroups	
	AUC	acc
Word2Vec U+V	95.66	63.17
GloVe U+V	96.34	65.06
E-U+V-0-F	96.76 (0.2)	65.86 (0.4)
E-U+V-u-F	96.79 (0.2)	66.30 (0.2)
E-U+V-ud-F	96.79 (0.4)	65.24 (0.6)
LE-U+V-0-F	96.65 (t3-w)	64.47 (t1)
LE-U+V-u-F	96.65 (t3-w)	64.54 (t1)
LE-U+V-ud-F	96.38 (t5-w)	64.76 (t3-w)

**Table 5:** Accuracy on test of IMDB Reviews sentiment analysis binary classification, with linear (BatchNorm + Dense) and with BiLSTM architecture (Bidirectional LSTM 32 channels, GlobalMaxPool1D, Dense 20 + Dropout 0.05, Dense), compared to baseline vectors. Best α and best limit method (on validation), are reported in parentheses.

Method	IMDB Reviews	
	acc lin	acc BiLSTM
Word2Vec U+V	82.84	87.61
GloVe U+V	83.76	88.00
E-U+V-0-F	83.58 (2.4)	88.12 (−4.0)
E-U+V-u-F	83.72 (−3.0)	88.56 (−4.0)
E-U+V-ud-F	84.23 (−3.0)	88.48 (−2.2)
LE-U+V-0-F	84.00 (t1)	88.36 (t1)
LE-U+V-u-F	84.29 (t1)	88.66 (t1)
LE-U+V-ud-F	84.00 (t3-w)	88.49 (t3-w)

**Table 6:** Accuracy of α-embeddings on test for the Stanford Natural Language Inference (SNLI) sentence entailment task, compared to GloVe and Word2Vec baseline vectors. We report experiments both with and without a projection matrix. The best values for α are reported in parentheses. The values presenting the largest improvement over the baselines are marked in bold.

Method	No Projection	Projection
GloVe U+V Word2Vec U+V	83.2 76.1	83.4 81.7
E-U+V-0-I	83.6 (−7)	84.2 (−4)
E-U+V-0-F	84.1 (−4)	84.2 (−1)
E-U+V-u-I	84.0 (−4)	84.0 (−4)
E-U+V-u-F	84.6(−8)	84.5(−8)
E-U+V-ud-I	83.8 (−1)	84.0 (−1)
E-U+V-ud-F	84.1 (−2)	84.5(−1)
GloVe U Word2Vec U	83.7 74.6	84.1 76.1
E-U-0-I	83.7 (+1)	84.1 (+1)
E-U-0-F	84.0(+3)	84.3(+1)
E-U-u-I	83.5 (−6)	84.0 (+1)
E-U-u-F	83.9 (−5)	84.2 (−10)
E-U-ud-I	82.8 (−6)	84.0 (+1)
E-U-ud-F	83.1 (−5)	84.0 (+1)
